# Yeast grown in continuous culture systems can detect mutagens with improved sensitivity relative to the Ames test

**DOI:** 10.1371/journal.pone.0235303

**Published:** 2021-03-17

**Authors:** Joseph Y. Ong, Julia T. Pence, David C. Molik, Heather A. M. Shepherd, Holly V. Goodson

**Affiliations:** 1 Department of Chemistry and Biochemistry, University of Notre Dame, Notre Dame, Indiana, United States of America; 2 Department of Biological Sciences, University of Notre Dame, Notre Dame, Indiana, United States of America; Centre National de la Recherche Scientifique, FRANCE

## Abstract

Continuous culture systems allow for the controlled growth of microorganisms over a long period of time. Here, we develop a novel test for mutagenicity that involves growing yeast in continuous culture systems exposed to low levels of mutagen for a period of approximately 20 days. In contrast, most microorganism-based tests for mutagenicity expose the potential mutagen to the biological reporter at a high concentration of mutagen for a short period of time. Our test improves upon the sensitivity of the well-established Ames test by at least 20-fold for each of two mutagens that act by different mechanisms (the intercalator ethidium bromide and alkylating agent methyl methanesulfonate). To conduct the tests, cultures were grown in small, inexpensive continuous culture systems in media containing (potential) mutagen, and the resulting mutagenicity of the added compound was assessed via two methods: a canavanine-based plate assay and whole genome sequencing. In the canavanine-based plate assay, we were able to detect a clear relationship between the amount of mutagen and the number of canavanine-resistant mutant colonies over a period of one to three weeks of exposure. Whole genome sequencing of yeast grown in continuous culture systems exposed to methyl methanesulfonate demonstrated that quantification of mutations is possible by identifying the number of unique variants across each strain. However, this method had lower sensitivity than the plate-based assay and failed to distinguish the different concentrations of mutagen. In conclusion, we propose that yeast grown in continuous culture systems can provide an improved and more sensitive test for mutagenicity.

## Introduction

Continuous culture systems allow for the long-term, controlled growth of microorganisms or cultured cells. Consequently, they have been used for many scientific and industrial uses, including producing biologics like small molecules [1–3] and recombinant proteins [4,5]; assessing the growth rate [6–9] or metabolism [10,11] of microorganisms or cultured cells under defined conditions; and for studying evolution [12–14]. Continuous culture systems typically operate in one of two modes: a chemostat, where a limited amount of nutrients are constantly added to the culture, or a turbidostat, where the culture density is kept constant [15]. Here, we present a test for using yeast grown in chemostats as a novel test for mutagenicity.

While analytical techniques, such as high-pressure liquid chromatography, can be used to detect the presence of mutagens, understanding the biological consequence of potentially mutagenic compounds requires assaying the compound against a biological sample. *In vitro* tests for mutagenicity typically expose bacteria or cultured mammalian cells to the suspected mutagen and assay for gross DNA damage, such as micronuclei or chromosomal aberrations, or for mutations of particular genes, such as HPRT with Chinese hamster ovary cells (reviewed by [16]). One of the common tests for mutagenicity is the Ames test, a bacteria-based test that uses reporter strains of *Salmonella typhimurium* to assess mutagenicity [17,18]. In the original form of this assay, the bacteria have a mutation in the histidine synthesis gene *hisG46* and are auxotrophic to histidine. The bacteria are exposed to the potential mutagen and plated in media with limited histidine, and the number of colony forming units (bacteria that mutated and reverted to being prototrophic to histidine) are counted and assessed as a measure of mutagenic potential. Similar assays have been developed using *Escherichia coli* and tryptophan synthesis [18,19]. Given the ease of use and short time needed to complete the assay, the Ames test can be performed in a high-throughput manner.

While inexpensive and easy to perform, the Ames test does have limitations. First, the Ames test exposes the bacteria to high concentrations of a single test compound over a short period of time, usually 48 hours [20], as low concentrations of mutagen may result in the control and experimental groups having similar numbers of mutant colonies, leading to an ambiguous result [21]. However, likely human exposure to suspected mutagens is expected to be at low concentrations over a very long period of time, such as when drinking water is contaminated by a mixture of pollutants, each present in very small concentrations [22]. In assessing the safety of such water, it is generally assumed that effects of potential mutagens are both linearly related to concentration and additive, but whether these assumptions are correct is difficult to determine with the present technologies [23]. Moreover, the Ames test and similar assays based on mutation reversion require that the strain be matched to the type of mutagen being detected: a strain with a point mutation is used to detect compounds (e.g., alkylating agents) that act by chemically changing DNA base pairs, while a strain with a frameshift is needed to detect intercalators [21,24]. An additional issue is that some compounds (e.g., the common DNA dye ethidium bromide) are only mutagenic after being metabolized, so mutagenicity tests with bacteria may require the addition of mammalian liver extracts (containing metabolic enzymes such as cytochrome P450) in order to reveal the mutagenic potential of the suspected compound [24,25]. Consequently, there is a need for an improved test for mutagenicity, one that has improved sensitivity and can assess multiple types of mutagens while remaining inexpensive.

Here, we present yeast grown in continuous culture systems as a novel method of mutagenicity testing. Yeast are an ideal organism for continuous culture systems because they grow robustly at room temperature (i.e., between 21–23 °C), are well-established model organisms, perform well in continuous culture systems [12,26], and have been previously functionalized as biosensors [27]. Moreover, as yeast are eukaryotes, their metabolisms and DNA repair pathways are more similar to those of humans, potentially providing an advantage for mutagenicity testing when compared to bacteria. Our systems are assembled using readily available materials standard to most microbiology laboratories, and our approach allows for the assessment of yeast exposed to mutagen over the span of approximately 20 days, permitting us to assay low concentrations of mutagen over long periods of time.

While our method is more time- and labor-intensive than the Ames test and consequently is less amenable to high-throughput applications (at least in its present form), our approach enabled us to detect levels of the mutagens methyl methanesulfonate (MMS; an alkylating agent) and ethidium bromide (EtBr; an intercalator) at concentrations at least an order of magnitude lower than the Ames test via a canavanine plate-based assay. Canavanine is a non-proteogenic amino acid structurally similar to arginine and toxic to wild-type yeast. Because yeast with loss-of-function mutations in the *CAN1* gene fail to import canavanine [28], the number of canavanine-resistant yeast colonies serves as a means of measuring mutagenic potential. We were also able to detect point mutations in yeast grown in MMS via whole genome sequencing, though with less sensitivity. Altogether, we demonstrate that a yeast-based chemostat approach is a viable method of determining the mutagenicity of compounds at low concentrations.

## Materials and methods

### Preparation and assembly of chemostats and associated equipment

Chemostats were constructed by drilling holes into standard 15 mL polypropylene conical tubes (VWR), with one hole at the bottom for the addition of an air inlet and one hole at the 5 mL mark for the addition of a waste outlet. Into the cap were drilled two adjacent holes for the addition of media. Ports and other fittings were purchased from Harvard Apparatus (PY2 72–1407, Polypropylene Luer Connector Kit). To the holes in the cap and at the bottom of the culture chamber were attached 1/16” barbed ports, and to the exit port was attached a 1/8” barbed port. These ports were sealed in place with commercial two-part epoxy (Loctite Epoxy Clear Multi-Purpose; we also used Imperial Hi-Temperature Silicone Sealant (red) KK0321) and allowed to set over 48 hours, per manufacturer’s instructions. One of the ports on the cap was sealed with a small piece of 1/16” tubing connected to a sealed plug. Prepared chemostats were placed in a sealed beaker and autoclaved. Autoclaving at 120 °C for 15 minutes showed no signs of degradation in the epoxy or elsewhere, even after repeated autoclave cycles.

To construct jars for media stocks, two holes were drilled through the caps of 500 mL Pyrex jars, and 1/8” female luers were attached through both of them and sealed with epoxy. One female luer was sealed with a male luer plug. Media was prepared (as described below), and 200 mL was transferred into the glass jar. The modified lid was placed over the jar and a length of 1/16” tubing was fed through the open female luer until the tubing comfortably reached the bottom of the jar. The opening of the female luer was closed with autoclave tape and aluminum foil. The media was then autoclaved at 120 °C for 15 minutes.

Peristaltic pumps (SP100 peristaltic pumps; APT Instruments, A/C, 15 rpm) were used to pump media into the chemostat. The inner tubing (silicon, 0.8 mm in diameter) was autoclaved for 120 °C for 15 minutes. The peristaltic pumps were plugged into a power strip that was controlled by an electronic outlet timer (Pixnor, Cat. No: ETU-63A), set to turn on the pumps for one minute every hour. This arrangement resulted in 0.40±0.1 mL of fresh media per hour (assessed by measuring the total volume delivered in ten feedings; n = 3 trials). An aquarium pump (Marina 200 Fish Tank Aquarium Air Pump) was used for aerating the chemostats. Before assembly, 0.22 micron filters (VWR, USA) were attached to the two outlets of the air pump to sterilize the air leaving the aquarium pump and entering the culture chamber. Each filter was connected with sterile 1/8” tubing to a four-way manifold. Four-way manifolds were sterilized with 70% ethanol and allowed to dry immediately before use. Stopcocks on the four-way manifold allowed for the adjustment of the airflow to a rate of 3.1±0.1 mL/minute (assessed by the length of time needed to displace 50 mL of water; n = 3 trials). The bubbling of the culture provided aeration and agitation in lieu of shaking.

Under these aeration conditions, we did not observe the sedimentation or flocculation of yeast, suggesting that the yeast cultures were adequately agitated and not severely oxygen-limited [29]. However, the amount of oxygen supplied under these conditions is probably well below the maximum oxygen consumption capacity: assuming 1% oxygen transfer per foot of depth [30,31], a 4 cm column of media, and a flow of 3 mL air/minute (corresponding to 1.48 mmol oxygen per hour), we estimate supplying 10% of the maximal oxygen consumption capacity (3.5 to 6 mmol of oxygen per gram dry weight of yeast [32,33]) for a culture of the size used here (0.003 g dry weight of yeast per 4 mL chemostat culture).

To assemble the culture chambers, the inner tubing of the peristaltic pumps and other tubing as described below were autoclaved. The inner tubing of the peristaltic pumps was positioned within the housing of the pump and secured. A length of 1/16” tubing about 30 cm long was used to connect the peristaltic pump to the cap of the culture chamber. Another length of 1/16” tubing was used to connect the bottom of the culture chamber to one of the outlets of the four-way manifold. To enable collection of waste or yeast samples, a length of 1/8” tubing was connected to the exit port of the chemostat, and the free end of the tubing was allowed to feed into a sterile 50 mL conical tube sealed with aluminum foil.

### Initiation and operation of the chemostats

To initiate the chemostats, a single colony of DBY10148 yeast [34] (*Saccharomyces cerevisiae*, gift of David Botstein), was first taken from a YPD plate and inoculated into 30 mL of synthetic media with ampicillin (described below) and grown at 30 °C with shaking at 250 rpm. After the overnight growth period (15–20 hours), this overnight preculture (OD 2.5–3.0) was diluted in synthetic complete media to an OD of 0.5. First, air flow was turned on and adjusted to a rate of 3 mL/minute. Then, culture chambers were filled with this diluted culture up to their exit ports (4.0 mL). After this step, peristaltic pumps were first turned on to allow the media to clear the empty space in the tubing, and then the pumps were allowed to operate on a feeding schedule of one minute per hour, as dictated by the electronic timer. The volume in the culture chamber during operation was 3.9–4.3 mL, with variation due to inconsistency in how much culture remained after excess culture flowed out during feeding. Chemostat cultures were maintained at room temperature (21–23 °C) for the designated time period. Whenever the media source was changed (e.g., because it was close to running dry), the pump corresponding to that chemostat was allowed to run until the media had cleared the empty space in the tubing and reached the culture chamber. A feeding rate of 0.4 mL/hourly feeding and a culture volume of 4 mL results in a dilution rate of 0.1 per hour.

#### Additional notes about chemostat operation

DBY10148 is a chemostat-adapted strain of yeast that is resistant to flocculating (clumping) or sticking to the sides of the continuous culture tube. Other strains could potentially be used, but for proper performance in the chemostats, it is imperative to avoid strains that clump and/or stick to the sides of the tubes. As noted below, our continuous culture systems are technically pulsed chemostats, not true chemostats, because they receive a small supply of media at fixed intervals instead of a continuous supply of media. For reasons of brevity we refer to them simply as chemostats.

### Preparation of synthetic media

Synthetic complete media was prepared by adding 20 g D-glucose (VWR, USA), 6.7 g Difco^™^ Yeast Nitrogen Base w/o Amino Acids (Bioworld, Grinnell, IA), and 2.0 g Drop-Out Mix Complete (US Biological, Swampscott, MA) to 1 L of DI water. The pH of the media was not adjusted and was 4.2. We used synthetic complete media instead of standard rich media (e.g. YPD) because clumping problems sometimes appeared when growing cultures in YPD. The solution was divided among five 500 mL jars (200 mL of media each). The media was autoclaved at 120 °C for 15 minutes and stored at room temperature until use. Ampicillin (Sigma, St. Louis, MO; final concentration 100 mg/L), methyl methanesulfonate (MMS; Sigma; used freshly prepared from stock bottle), and ethidium bromide (EtBr; Fischer, Fair Lawn, NJ; aqueous stock solution: 10 mg/mL) were added through the screw-cap luer port on the media jar lid before use. Aluminum foil was wrapped around media containers to protect EtBr from degradation from light, and media was stored away from direct sunlight.

### Preparation of canavanine plates

Canavanine plates were made by adding 20 g D-glucose (VWR), 6.7 g Difco^™^ Yeast Nitrogen Base without Amino Acids (Bioworld), 2.0 g Drop-Out Mix Synthetic Minus Arginine without Yeast Nitrogen Base (US Biological), and 20 g agar (Research Products International (RPI); Mt. Prospect, IL) to 1 L of DI water and autoclaving at 120 °C for 15 minutes. When the mixture had cooled to about 55 °C, canavanine sulfate (Sigma; final concentration: 30 mg/L) and ampicillin (Sigma; final concentration: 100 mg/L) were added and the mixture allowed to stir before pouring into 15 x 100 mm plates (about 25 mL per plate). Plates were allowed to set overnight and stored at 4 °C until use.

### Canavanine plate assay

To collect a yeast sample, the exit port was switched to feed into a sterile 15 mL conical tube and left for 2–3 hours to collect about 1–2 mL of yeast culture. Five hundred microliters of the collected yeast culture were saved as a glycerol stock (25% by volume glycerol) and stored at -80 °C. The culture density (OD600) was measured and 1 OD unit of yeast was plated on canavanine plates. Lower amounts of yeast (e.g., 0.5 OD units) were plated as the number of canavanine-resistant colonies increased to maintain evenly spaced and countable colonies, and the reported values were adjusted to account for this dilution. Sterile PBS was used to bring the volume of yeast up to 200 μL if necessary and the yeast culture was spread on the plate with sterile glass beads. Each plating was done in triplicate. The plates were incubated at 30 °C for two or three overnights, after which canavanine-resistant colonies were manually counted. The source data, including the number of counted canavanine-resistant colonies for all experiments, are available in S1 Table. To obtain the relative normalized average, the number of canavanine-resistant colonies obtained in triplicate for each chemostat were averaged and presented relative to the no mutagen control. To obtain the relative normalized standard deviation, first, the standard deviation of the number of canavanine-resistant colonies obtained in triplicate for each chemostat was calculated. Then, the ratio of the standard deviation to the raw (non-normalized) average was applied to the relative normalized average to calculate the normalized standard deviation. Yeast samples were usually collected every two to three days for freezing and plating.

A Tukey-HSD (honestly significant differences) test was used to determine the statistical significance in R. For each time point, the number of canavanine-resistant colonies from yeast exposed to mutagen were compared to the number of canavanine-resistant colonies from the no mutagen control.

### Yeast DNA extraction

For yeast genomic DNA extractions, we adapted a protocol from previously described approaches [35]. Details of our protocol are available online: https://dx.doi.org/10.17504/protocols.io.bptbmnin. Briefly, yeast from frozen glycerol stocks were streaked onto YPD plates and used to inoculate 30 mL of YPD and grown at 30 °C overnight with shaking at 250 rpm. The yeast cell wall was digested with Zymolyase. The resulting yeast spheroplasts then underwent an alkaline lysis, and, after clearing the lysate with potassium acetate and centrifugation, the resulting genomic DNA was isolated and purified by an RNase A treatment and alcohol precipitations.

### Counting variants via whole genome sequencing

Sequencing of DNA samples as prepared above was performed at the University of Notre Dame Genomics and Bioinformatics Core Facility with an Illumina MiSeq desktop sequencer. Twenty-seven genomes from yeast grown in MMS as in Fig 2A (specifically the starting strain of yeast (Day 0) and the Day 9 and Day 18 time points from all four mutagen conditions, each in triplicate) were prepared with an Illumina Nextera DNA Sample Preparation Kit with V3 600 cycle kit to generate 300 base pair paired-end reads. Reads were processed through the University of Notre Dame Center for Research Computing. The CEN.PK2-1 reference genome [36] (GenBank JRIV00000000.1) or S288C reference genome (GenBank GCA_000146045.2) was indexed with BWA [37] (version 0.7.17), and Illumina reads were aligned to the reference genome to generate SAM files. SAM files were converted to BAM files, and BAM files were sorted and indexed using SAMtools [38] (version 1.9).

For variant calling with VarScan2, an MPILEUP file was generated with SAMtools. Variants were called with VarScan2 [39] (version 2.4.3) using somatic mutation calling. One colony of the initial culture of yeast served as a baseline (“normal”) to which each colony of yeast grown in continuous culture was compared (“tumor”). Single nucleotide polymorphisms (SNPs) were manually filtered to exclude ambiguous calls (that is, SNPs where two different alleles were called at one genomic position) and to exclude SNPs with a p-value greater than 0.05 (as determined by VarScan2 via Fisher’s Exact Test). For variant calling with Mutect2, a dictionary for the CEN.PK2-1 reference genome was created with Picard tools (https://github.com/broadinstitute/picard, version 2.22.4), and the reference genome was indexed using SAMtools. Read groups were added to sorted BAM files with Picard tools. One of the Day 0 colonies, designated as the baseline (“normal”) was given sample ID 1, and all other colonies were each compared to this baseline colony. To call variants, Mutect2 from GATK [40] (version 4.1.7.0) was used in a similar manner as VarScan2 to call variants. As above, SNPs were manually filtered to exclude ambiguous calls and to exclude SNPs with 2 or fewer reads supporting that call.

### Calcofluor white staining

A glycerol stock of DBY10148 was streaked out onto a YPD plate, and a single colony was used to inoculate 30 mL of synthetic complete media. The yeast culture was allowed to grow at 37 °C for 16 hours with shaking at 250 rpm. 100 μL of the late log-phase culture (OD600 = 2.85) was centrifuged at 180 x g for 1 minute to pellet the yeast and resuspended in 40 uL of a solution containing a 1:1 ratio of calcofluor white staining solution (Sigma, Cat. # 18909) and 10% KOH (w/v) as per manufacturer’s instructions. The yeast suspension was incubated in the dark for 1 minute before transferring onto a microscope slide and imaging. Yeast were imaged using a Nikon Eclipse Ti2 with 60x, 1.4 N.A. objective. Images were obtained by a Hamamatsu CMOS camera controlled by NIS-Elements BR 413.04 64-bit software (Nikon). Data were further processed using FIJI (National Institutes of Health).

## Results

### Construction of chemostat devices

**Fig 1:**
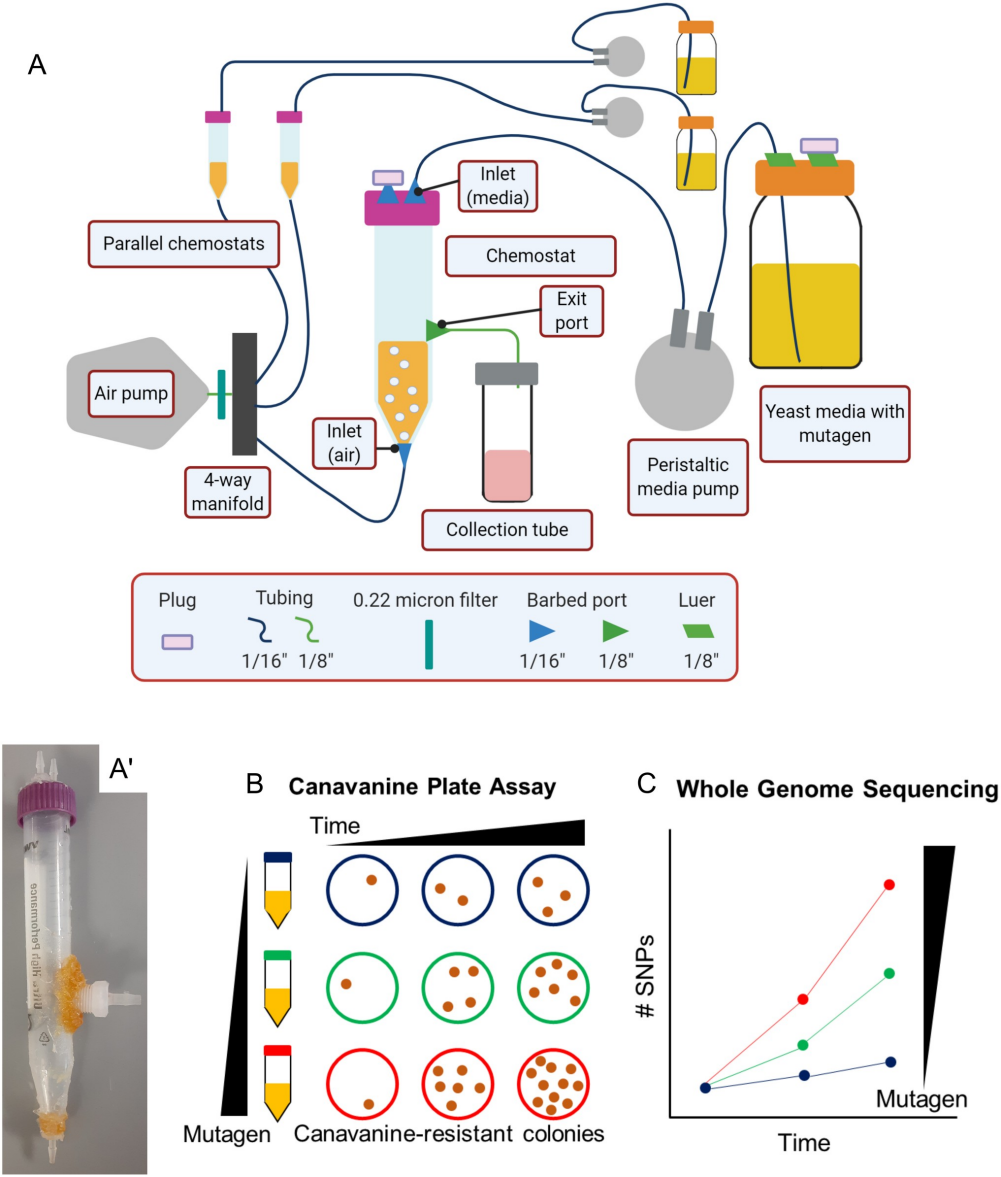
Overview of approach for using yeast as a biosensor for mutagenicity. (A) Scheme of a chemostat culture system. Note the positions of inlet ports for aeration and media input and the outlet port for collecting waste and yeast samples, as well as allowing air exhaust. While typically four chemostats were run in parallel, only three parallel chemostats are shown here for clarity, with the central chemostat shown in detail. (A’) Photograph of one of the prepared chemostats from (A). The length of the 15 mL conical tube is 12 cm. (B) Scheme of the canavanine-based plate assay, where the number of canavanine-resistant yeast colonies serves as a measure of mutagenic potential. (C) Scheme of the whole genome sequencing-based assay, where the number of single nucleotide polymorphisms (SNPs) or other mutations serves as a measure of mutagenic potential.

We designed an inexpensive continuous culture system that would allow yeast to grow undisturbed over a long period of time (weeks). For the growth chamber, we used a conventional 15 mL conical tube (Fig 1A’ and 1A”) modified as described in the Methods section. Briefly, in our cultures, fresh media was delivered by a timer-driven peristaltic pump, and the culture was both mixed and aerated by bubbled air. To keep the volume of the culture constant, excess culture and media were removed by gravity via an exit port (Fig 1A’). Because media was provided at regular times instead of continuously, our culture systems are technically “pulsed chemostats” instead of true chemostats, though we refer to them as chemostats below. We opted for a pulsed instead of continuous media delivery to keep the costs of our systems low.

To run a set of chemostats, an overnight culture of yeast was first diluted down to 0.5 OD600, and each culture system was filled to its exit port. A timer then turned on the media peristaltic pump for one minute every hour, replacing approximately 1/10 of the culture volume at each feeding (see Methods for more information). In a chemostat, the growth rate is defined by the rate of dilution; in our system, the dilution rate was 0.09–0.11 per hour, meaning each culture doubled approximately every 10 hours. This dilution rate (which is much slower than the approximately 2 hour doubling time of DBY10148 in synthetic media) was chosen to maximize the growth rate while promoting culture stability (that is, preventing the culture from being “washed out” by excess media, as will occur if the culture dilution rate gets too close to the log phase growth rate [41]). In each run of the experiment, four or five of these cultures, each with a different concentration of mutagen, were run in parallel.

### Use of yeast chemostats to measure mutagenicity of the alkylating agent MMS

**Fig 2:**
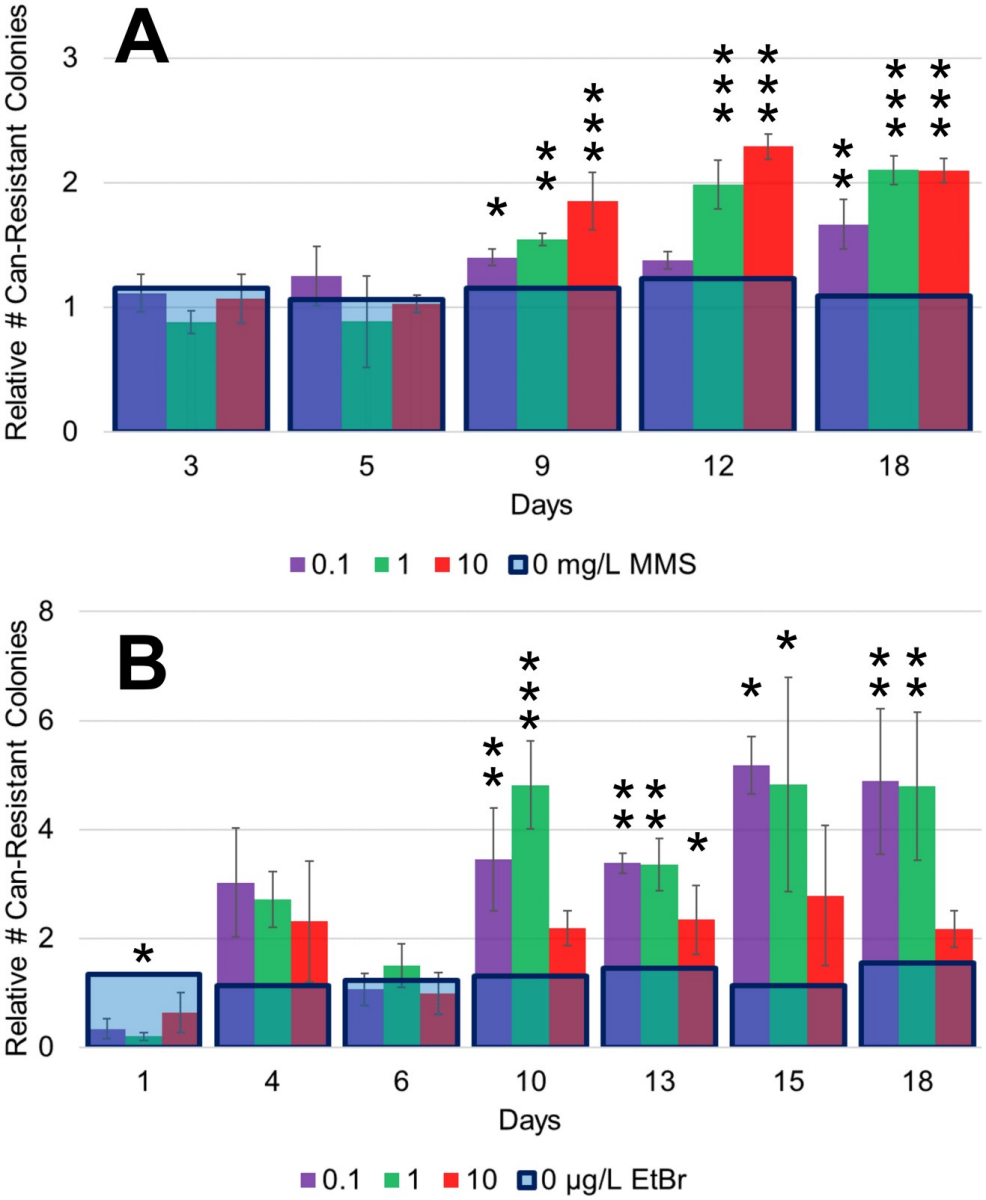
Combining chemostat growth and a canavanine plate assay enables improved mutagen detection. Yeast were grown in parallel chemostats over three weeks in varying concentrations of the methylating agent MMS (A) or DNA intercalator EtBr (B) and plated on canavanine plates. The number of canavanine-resistant (Can-resistant) colony forming units was counted after 48 hours and graphed as fold-increase (± standard deviation) relative to the number of Can-resistant colonies from the yeast grown with no mutagen. Each yeast strain was plated in triplicate at each time point. The blue box represents the normalized average for the number of Can-resistant colonies in the no mutagen control (that is, 1) + normalized standard deviation. The Ames test limits of detection are 2.2–4.4 mg MMS/L [43,44] and 2.0 μg EtBr/L [21]. A Tukey-HSD test was used to determine statistical significance (* = p < 0.05, ** = p < 0.01, *** = p < 0.001).

To assess the ability of these chemostats to detect lower concentrations of mutagens, we first focused on testing the DNA methylating agent methyl methanesulfonate (MMS) [42]. Given that the limit of detection for the Ames test is approximately 2.2–4.4 mg MMS/L [43,44], we began by growing yeast in four parallel chemostats with 0, 0.1 1.0, and 10 mg MMS/L (Fig 2A). Our lowest concentration of MMS tested, 0.1 mg MMS/L, corresponds to 22 times lower than the Ames test detection limit. To assess the mutagenicity of each level of MMS, we took samples of yeast from each chemostat at regular intervals over the period of a month. We then plated them on synthetic media plates without arginine and supplemented with canavanine, using the number of canavanine-resistant yeast colonies as a measure of mutagenicity (Fig 1B).

We observed that the number of canavanine-resistant colonies increased with time and with the concentration of MMS, although not all time points were equally suitable for assessing the level of mutagen. First, it was not possible to distinguish different levels of mutagen until the second week of growth, even though the culture reached its steady-state OD within 48 hours. This delay is not surprising because time is needed for mutations to accumulate in the population. Second, the data became noisy after a period of about 18 days, obscuring the difference between levels of MMS. We speculate that this increased noise is because of evolution occurring within the population [12,26]. For example, if a cell that is wild-type at the canavanine locus acquires a mutation that enables it to compete significantly better for nutrients in the chemostat environment, then the number of observed canavanine-resistant colonies will drop below that expected for the given level of mutagen. This is a problem that is expected to become more significant with time.

Thus, we concluded that the data from the yeast chemostats were most accurate from one to three weeks of growth. For this reason, we present the interpretable portion of the data (up to Day 18) in the main text but have provided the full time course in the supplement (S1 Fig). Moreover, we concluded that we could detect MMS as low as 0.1 mg MMS/mL, about 20 times lower than the Ames test, and that the relationship between the mutagenicity of MMS and its concentration is direct in the range tested, including below the Ames test limit of detection.

### Use of yeast chemostats to measure mutagenicity of the intercalating agent EtBr

To further validate our system, we next grew yeast in the presence of the DNA intercalator ethidium bromide (EtBr). Reported values for the Ames test detection limit of EtBr range from 2.0 μg EtBr/L (in the presence of mammalian liver extract) to >20,000 μg/L (in the absence of mammalian liver extract; EtBr becomes toxic before its mutagenicity is detectable when the extract is not present) [21]. We grew yeast for a period of three weeks at 0, 0.1, 1.0, and 10 μg EtBr/L and plated them on canavanine plates as described in Methods (Fig 2B). Our lowest concentration of EtBr tested, 0.1 μg/L, corresponds to 20 times lower than the Ames test detection limit. Once again, each condition gave a distinguishable number of canavanine-resistant colonies relative to the untreated control after about a week. Interestingly, we observed that the lower concentrations of EtBr (0.1 and 1.0 μg EtBr/L) produced higher numbers of canavanine-resistant colonies than the highest concentration of EtBr (10 μg EtBr/L), a biphasic response consistent with hormesis. Briefly, hormesis is when a low dose of a toxin produces a stronger effect than a higher dose, perhaps because the higher dose induces protective responses not triggered by the lower dose [45,46]. This observation was reproducible in two more independent runs of the parallel chemostats (S1C and S1D Fig).

We wondered if toxicity of EtBr could account for the observation, even though the concentration of EtBr we used here is far lower than the concentration observed to induce toxicity in *S*. *typhimurium* (20,000 μg EtBr/L) [21]. To assess the growth of the yeast exposed to EtBr, we tracked culture density and the number of colony forming units on YPD (that is, in the absence of mutagen or canavanine) over time. We saw no significant differences in the culture OD (S2A Fig) in chemostats exposed to EtBr relative to the untreated control chemostat. We saw a minor difference in the number of colony forming units per OD (S2B Fig) in the yeast exposed to EtBr relative to untreated control yeast. However, this difference was not statistically significant. These observations are consistent with the conclusion that the levels of EtBr assayed here were not cytotoxic.

In conclusion, similar to MMS, the chemostats could reliably detect the mutagen EtBr as low as 0.1 μg/L, 20 times more sensitive than the Ames test detection limit of 2.0 μg/L (though the chemostats failed to detect the mutagen at 0.01 μg/L, S1C and S1D Fig). Moreover, unlike the direct relationship seen with MMS, our data provide evidence for hormesis in EtBr. In other words, our data suggest that lower concentrations of EtBr (below those detectable by the Ames test) may have higher mutagenic potential than higher concentrations of EtBr.

### Use of whole genome sequencing to assess the number of mutations

Next, we asked whether we could use whole genome sequencing as a more sensitive measure of mutation rate. By sequencing and analyzing the whole genomes of individual yeast colonies, we hypothesized that we could get a more sensitive and direct readout of mutagenicity (Fig 1C) than from assessing only mutations in a single gene (the *CAN1* locus), as occurs in the plate assay (Fig 1B). As shown below, our hypothesis was not correct, at least given the number of colonies we sequenced and the depth of sequence coverage that we used. We present these data and our analysis here so that they may be useful to others attempting to follow up on this work.

From glycerol stocks of yeast grown in the MMS cultures, we isolated the DNA from three independent colonies of yeast grown in chemostats for 9 and 18 days for all four levels of mutagen. We chose day 9 because it was the first day where the canavanine plate data could detect a difference among the different levels of mutagen (that is, we thought samples from day 9 would be the earliest time point from which we would have obtained interpretable sequencing data), and we chose day 18 because it was the last time point before the canavanine plate data became difficult to interpret due to increased noise (as noted earlier, we speculate this increased noise comes from evolution of the cultures over time). We also isolated DNA from three colonies of the yeast used to initially seed the chemostat (“day 0”). We then sequenced the genomes of these yeast for a total of 27 genomes at approximately 10x coverage (see Methods). We originally aligned our reads against the *S*. *cerevisiae* reference genome for strain S288C (a standard *S*. *cerevisiae* laboratory strain) or CEN.PK2-1 (the parental strain for our sequenced DBY10148 strain) and used traditional variant calling tools like GATK or VCFtools to identify variants. With this method, however, the number of variants correlated with the number of reads per strain, suggesting that differences arising from inequalities in alignment and coverage were the main contributors to the number of SNPs detected. Moreover, most of the variants detected were differences between our DBY10148 strain and the reference genome strains. To address this issue, we tried generating a *de novo* reference genome using Jellyfish (version 2.2.1) [47] and our Day 0 seed strains, but the coverage of the assembled reference genome was poor, leading to artifacts in data analysis.

**Fig 3:**
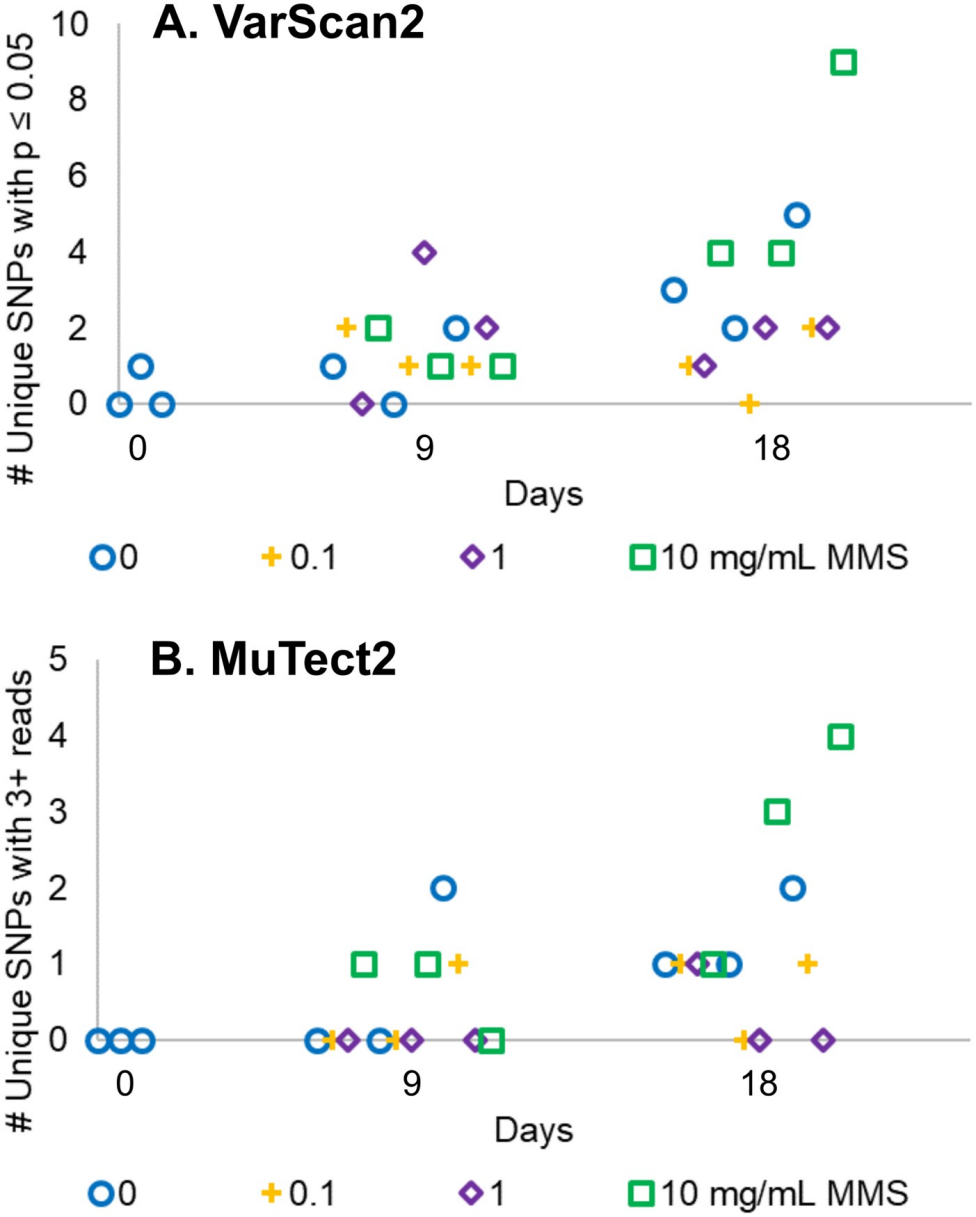
Somatic mutation calling can identify SNPs but not distinguish yeast grown in differing levels of mutagen. Genomic DNA from 27 individual yeast colonies (from Day 0, 9, and 18) from Fig 2A were sequenced and the number of SNPs quantified, relative to one of the yeast colonies grown in no mutagen at Day 0 using either (A) VarScan2 or (B) Mutect2. Identified SNPs were filtered to remove ambiguous SNPs (SNPs with more than one allele at given genomic position) and those that did not pass a quality check (for VarScan2, p value ≤ 0.05 by Fisher’s exact test; for Mutect2, only SNPs with 3 or more reads in “normal” and “tumor” were considered).

To improve our SNP quantification method, we employed somatic mutation calling. Somatic mutation calling, a technique traditionally used to compare sequences from matched tumor and normal tissue samples, compares aligned reads from one sample to another. This technique bypasses inequalities in coverage, as a comparison is only made if both reads have sufficient coverage at a particular locus. Single nucleotide polymorphisms (SNPs) were identified using the somatic mutation caller VarScan2 (Fig 3A) [39]. We used one colony of the initial seed culture as the “normal” sample and one of the remaining 26 genomes as “tumor” samples to identify SNPs caused by MMS exposure and time.

Of the approximately 5,000 SNPs identified, the large majority (~90%) were variant calls with two or more alleles per genomic position, as if the yeast were diploid. This was surprising because DBY10148 is a MATa haploid strain [34]. Calcofluor white staining of late log-phase cultures of DBY10148 showed axial bud scars at only one end (S3 Fig), further supporting the conclusion that the DBY10148 strain indeed is haploid [48]. To understand why sometimes multiple alleles were found at a single genomic position, we analyzed the location and sequence of our diploid-like variants relative to the *S*. *cerevisiae* S288C reference genome (GenBank GCF_000146045.2; the S288C reference genome is better annotated than the CEN.PK2-1 genome, allowing for easier analysis of SNP location). We saw that these diploid-like variants clustered together (S4 Fig). The locations of these diploid-like SNPs corresponded to repetitive DNA sequences like telomeric DNA and viral transposons, similar to what has been previously reported [49]. We reasoned that the alignment method could not accurately map reads corresponding to repetitive DNA sequences [50] and thus disregarded these diploid-like SNPs.

The remaining approximately 500 SNPs that mapped to only one unique genomic location were analyzed to identify variants between the “normal” initial seed culture and the “tumor” mutagenized yeast cultures. Only SNPs that varied between the “tumor” and “normal” with a p-value of 0.05 or lower were counted. Through this process, we identified a number of SNPs that arose due to the spontaneous generation of mutations over time and/or due to the mutagen MMS. However, the number of SNPs identified in yeast cultures grown in any level of mutagen was not distinguishable from yeast grown in no MMS (Fig 3A). As MMS was expected to cause SNPs (and not base pair insertions or deletions [InDels]), we focused our analysis up to this point on quantifying the number of SNPs. A parallel analysis of InDels also demonstrated no significant difference in the number of InDels between each sample (S5 Fig).

To further investigate the mutations in our chemostats, we manually analyzed the SNPs identified with VarScan2 to determine the consequence of each SNP. Each SNP was classified as either falling in an intergenic region (that is, between two coding regions), or, if the SNP was located within a protein-coding open reading frame, whether the SNP caused silent, missense, nonstop (stop codon to amino acid), or nonsense (amino acid to stop codon) mutations. For each SNP falling within a coding region, we also identified the gene. These results are summarized in S2 Table and visually in S6 Fig.

These data show that almost half of the mutations (21/55) were in regions categorized as intergenic. Of the mutations in coding regions (34), approximately 60% (20/34) were silent and 14 were missense. We did not identify any nonsense or nonstop mutations. Across the 27 genomes, we identified 21 genes that were mutated (S2 Table). These genes were not enriched for a particular biological process (determined via Gene Ontology term analysis) [51], and with one exception, each of these genes was found to be mutated in only one of the 27 genomes sequenced. We did not detect any mutations in the *CAN1* gene. This was not surprising given that most yeast in our experiments (including those used for sequencing) were never exposed to canavanine: only small samples of the yeast leaving the chemostats were plated onto the canavanine containing plates. Thus, the yeast that we sequenced never experienced positive selection for mutation of the *CAN1* gene.

At first glance, these data are consistent with the hypothesis that most of the observed mutations are neutral. However, there is some evidence for selection occurring in our chemostats. Most significantly, the gene *ENO1* (coding for the glycolytic enzyme enolase/phosphopyruvate hydratase) was mutated in three of the 27 sequenced colonies: one corresponding to 1 mg MMS/L at Day 9, one corresponding to 1 mg MMS/L at Day 18, and one corresponding to 10 mg MMS/L at Day 18. As these mutations were all unique (that is, we did not detect shared mutations that would suggest that the mutations were passed on from mother to daughter cell), we believe these mutations occurred independently. We were unable to determine the consequence of these mutations on the function of the enzyme, as they did not occur at obviously important residues, such as amino acids in the active site. However, the repeated mutation of enolase, an important enzyme in glycolysis and consequently an important regulator of metabolism within the yeast cell, is evidence of genetic selection for yeast with metabolic profiles that positively select for growth in a chemostat. Consistent with this hypothesis, previous work has shown that lower expression of *ENO1* led to increased fitness in batch-culture growth assays [52] and in glucose-limited chemostat cultures [53]. These *ENO1* mutations support our speculation that evolutionary forces (e.g., clonal selection) might be causing the noise observed in the canavanine plate assay after about 18 days in culture (S1 Fig).

To confirm the inconclusive relationship between the number of SNPs observed and the level and time of exposure to mutagen, we repeated the same analysis with Mutect2, another somatic mutation calling algorithm (Fig 3B). The results using Mutect2 were similar to those from VarScan2, suggesting that our results are not due to bias within the somatic mutation calling program used. Altogether, the sequencing data indicate that further optimization would be necessary in order to use whole genome sequencing as a means of assessing mutagenic potential.

## Discussion

Continuous culture systems allow for the growth of a microorganism culture over a long period of time. Here, we demonstrate that yeast grown in chemostats can be used as an improved biosensor for mutagenicity of a test compound in aqueous solution that is supplied with the growth media. These chemostats were operated for a period of up to a month in the presence of mutagens at different concentrations; as discussed more below, they yielded useful mutagenicity data in the time period between 1 week and 18 days. The mutagenicity of the test compounds was then quantified by plating samples of the cultures on canavanine plates, counting the number of colonies that appeared, and comparing this number to yeast cultured in media without mutagen. Using this assay, we were able to detect the effects of two established mutagens (the DNA methylating agent MMS and DNA intercalator EtBr) at levels more than one order of magnitude lower than the Ames test. Thus, our yeast assay enables more sensitive detection of mutagenicity of test compounds as well as assessment of the effects of long-term exposure to low levels of established mutagens. Moreover, these devices are relatively cheap and inexpensive to operate, requiring mostly standard laboratory equipment and reagents, such as agar plates.

However, compared to the Ames test, our assay does require more time and labor. Whereas the Ames test can be performed in a fast and high-throughput manner, being able to screen through compounds in a matter of days, our devices require more bench space, more manual work (in terms of collecting, plating, and freezing yeast), and a longer assay time, requiring a number of weeks to characterize one compound. Compared to the Ames test, the advantage of our continuous culture assay is its greater sensitivity and thus its ability to study the mutagenic effects of compounds below the Ames test limit of detection. We envision our system may also have an advantage compared to the Ames test when testing combinations of untested or unknown compounds, such as low-level pollutants or trace chemicals present in reclaimed or environmental water samples.

The increased sensitivity of our test enabled us to investigate the relationship between the concentration of mutagen and the level of observed mutagenicity at concentrations below the Ames test limit. Typically, this relationship is assumed to be direct, but whether this assumption is accurate has been difficult to assess [54–56]. We observed that as the concentration of MMS increased, the number of canavanine-resistant colony forming units increased in a direct manner (Fig 2A), consistent with expectation. However, as the concentration of EtBr increased, the number of canavanine-resistant colony forming units from yeast grown in 10 μg EtBr/L was fewer relative to yeast grown in lower concentrations (0.1 or 1.0 μg EtBr/L) (Fig 2B). This observation was surprising.

To explain the observation that a higher concentration of the EtBr mutagen resulted in fewer mutations, we first hypothesized that the EtBr might have a toxic or growth-inhibiting effect. However, we detected no obvious growth defects in cultures grown in EtBr as assessed either by optical density of the continuous culture or by the number of colony forming units on YPD (with no mutagen or canavanine) (S2 Fig). We cannot rule out that the highest concentration of EtBr tested may have had an undetected toxic effect on the yeast that would explain our biphasic results. One possible explanation is that higher concentrations of EtBr may have triggered a defense mechanism, such as efflux of EtBr or upregulated DNA repair pathways, that may explain the lower number of canavanine-resistant colonies. Such an explanation would be consistent with hormesis [45,46]. Regardless, the number of canavanine-resistant colonies from yeast grown in EtBr from 0.1 to 10 μg EtBr/L was significantly more than control (Fig 2B), demonstrating that we could detect the mutagenic potential of the compounds tested even at concentrations lower than the limit of detection for the Ames test.

Importantly, while analytical techniques like HPLC can detect the presence of potential mutagens at lower concentrations than we assayed here, the biological effects of those compounds cannot be analyzed without a biological system. In particular, analytical techniques cannot determine whether an unknown or uncharacterized compound is mutagenic or genotoxic, nor can it inform about the dose-response relationship. For example, the observation that the relationship between mutagen concentration and mutagenicity is direct for MMS but hormesis-like for EtBr is one that could not have been directly inferred from simply detecting the amount of mutagen present via HPLC. While such biological assays are more time-consuming and involved than simpler analytical chemical assays, this observation highlights the importance of using biological systems to understand the biological consequence of mutagen exposure.

### Potential improvements

Variants of the Ames test typically use two additional elements to improve the sensitivity and accuracy of their results. First, the test can be performed with and without the addition of S9 liver extract, as some compounds are not mutagenic but their metabolites or derivatives are [17,25]. Secondly, the typical bacteria used in the Ames test have defects in their DNA repair pathways, allowing for greater sensitivity. While we did not use either of these two elements in our yeast-based system, these may be possible avenues for the improvement of our yeast-based tests for mutagenicity. Importantly, we note that whereas bacteria-based assays require the addition of S9 liver extracts to detect the mutagenic potential of EtBr [21], our yeast-based assay was able to detect the mutagenic potential of EtBr without S9 liver extracts, suggesting that the eukaryotic metabolism of yeast may serve as an advantage for using yeast as a biosensor for mutagenicity.

Our cultures operated at a dilution rate of approximately 0.1 per hour. We opted for this dilution rate as a reasonable compromise between fast, active growth and maintaining the stability of the culture. At faster dilution rates, we were concerned that yeast would not grow fast enough and the culture would dilute or wash out over time [41]. Testing how variations of the dilution rate affect culture performance is a potential avenue for future study. In a similar manner, the oxygen consumption rate is a factor that regulates the metabolism of yeast, particularly with regards to redox balance [57] and respiration [58]. While we bubbled air through the culture at approximately 3 mL/minute (providing 1.48 mmol of oxygen gas through the chemostat per hour), we made no measurements as to the oxygen consumption rate or metabolic state of the yeast. Consequently, though we did not measure this directly, it is likely that the yeast in our chemostat were at least somewhat oxygen-limited (see Methods section for details). Oxygen limitation could potentially affect the rate at which compounds are metabolized. Testing oxygen levels within the chemostat or the effects of altered oxygen delivery may be additional future directions, especially for cases where the metabolism of a potential mutagen is expected to involve redox processes. On the other hand, our goal was to make the assay as inexpensive and straightforward as possible, and additional measurements and/or environmental controls would increase cost.

### Whole genome sequencing as an attempted improvement to the canavanine plate assay

One potential improvement that we did attempt was to assess mutagenic potential via whole genome sequencing. We were originally interested in the sequencing-based approach to quantifying mutagenicity in the continuous cultures because of its potential to be scaled up and automated. While whole genome sequencing was able to detect SNPs in our samples, we observed that the sequencing approach was not as sensitive as the plate-based assay. The data were difficult to interpret for two main reasons. First, for cost reasons, we sequenced at only 10x coverage, and at this coverage and replicate number it was difficult to distinguish SNPs from sequencing errors. Second, the presence of repetitive DNA sequences within the yeast genome made some read alignments ambiguous. Consequently, when a SNP emerged in a repetitive sequence of DNA, it was difficult to distinguish a genuine variant from an artifact of poor alignment. As a result, the overall coverage of our genomic sequences was further reduced because some of the repetitive reads were discarded. In addition, because of cost limitations, we were only able to sequence three individual yeast colonies per condition, which further limited the scope of our analysis.

In retrospect, one might have predicted that the plate-based approach toward determining mutagenicity would be more sensitive than the sequencing approach because each plate test assays ~10 million copies of the 1773 base pair *CAN1* gene (the 1 OD unit applied to each plate is approximately 1E7 cells, and many different mutations in the 1773 base pair gene can result in canavanine resistance). In contrast, each genome sequence provides information on only the ~12 million base pairs of the yeast genome. A sequencing approach would be strengthened by increasing the read depth, the number of colonies sequenced, and the number of time points sampled, as more data points are needed to obtain the resolution necessary to distinguish between the different concentrations of MMS. However, sequencing costs would increase. Moreover, improved methods of mapping repetitive DNA regions may improve the sensitivity of assessing mutagenicity by sequencing. Thus, practical application of the sequencing approach may await further improvements in sequencing technology and bioinformatics.

One benefit of the sequencing analysis that we performed is that it provided a potential explanation for the noise observed in the canavanine plate assay about 18 days in culture. As noted above, the observation that *ENO1* mutations were observed multiple times indicates that evolution was occurring in the cultures. This might contribute to the noise observed at later time points (S1 Fig) in the following way: if an advantageous mutation occurred in a cell that lacks canavanine resistance, expansion of that clone at the expense of other cells would artificially suppress the level of canavanine resistance. The opposite would be true if such an advantageous mutation occurred in a cell that already had canavanine resistance. This expansion of one clone at the expense of other cells in the population is known as clonal interference [59], and it would be expected to increase the variability of the results of the canavanine plate assay. Exactly when this variability would be expected to become problematic is not yet well-defined, but the observation of increased noise at 18 days (S1 Fig) suggests that these factors become problematic after this point. Moreover, as we assume that when each mutation occurs is random, at which time point a mutation occurs may not be the same between different runs of parallel chemostats. We suggest that the variation in when advantageous mutations appear also contributes to the observed variability between chemostat runs.

One reason that we remain interested in the sequencing-based approach for assessing accumulated mutations (and thus mutagenicity) is that has potential to overcome the clonal interference problem observed at long time points because neutral mutations should silently accumulate in the background of any strain. In the meantime, the plate assay remains straightforward and practical for exposure times up to approximately 20 days and provides a roughly 20x improvement over the sensitivity of the Ames test for at least two mutagens tested, MMS and EtBr.

## Conclusions

In conclusion, we present a method for using the long-term continuous culture of yeast as the foundation of an improved biosensor for mutagenicity. We have shown that this biosensor works for two mutagens that act by different mechanisms (MMS, DNA methylation; EtBr; DNA intercalation) and suggest that it might have particular utility for assessing the safety of compounds (e.g. food additives, trace contaminants in reclaimed water) to which humans are exposed at low concentrations for long periods of time.

## Supporting information

S1 Fig Complete timecourses and replicates for yeast grown in chemostats. (A) Complete timecourse of MMS chemostats shown in Fig 2A. (B) Complete timecourse of EtBr chemostats shown in Fig 2B. (C, D) Two additional replicates of EtBr chemostats performed in a similar manner to Fig 2B. While yeast grown in chemostats with 0.1, 1.0, and 10 μg EtBr/L consistently produced more canavanine-resistant colonies compared to the no mutagen control, the concentration 0.01 μg EtBr/L was unable to be reliably detected. Moreover, the concentration 1.0 μg EtBr/L consistently produced more canavanine-resistant (Can-resistant) colonies than 10 μg EtBr/L. Data are presented as in Fig 2. A Tukey-HSD test was used to determine statistical significance (* = p < 0.05, ** = p < 0.01, *** = p < 0.001).
(TIFF)

S2 Fig Low levels of EtBr do not cause toxicity to yeast grown in continuous culture systems. (A) To determine if EtBr had significant toxicity to the yeast, the OD600 of the yeast was assessed over time to identify growth defects and was determined to have no significant variation. (B) To determine if there was any difference in the number of colony forming units, 1.25E-6 OD units of yeast (roughly 40 cells) were obtained by serial dilution and plated onto YPD plates in triplicate. The number of colony forming units (CFU) was counted after 48 hours at 30 °C and is presented as the average of the three trials. The error bars represent standard deviation. The data here correspond to the EtBr chemostats presented in S1D Fig.
(TIFF)

S3 Fig DBY10148 yeast exhibit axial bud scars. A colony of DBY10148 was used to inoculate an overnight culture of synthetic complete yeast media and allowed to reach late-log phase (OD600 between 2.5 and 3 after about 17 hours of growth at 30 °C). The culture underwent staining with calcofluor white to visualize bud scars. Bud scars were categorized as being either axial (at one pole, expected for haploid yeast) or distal (at both poles, expected for diploid yeast). (A) and (B) are representative images of axial bud scars observed in 96.7% (148/153) of yeast cells analyzed. (C) is a distal bud scar pattern representative of 4.3% (5/153) of yeast cells analyzed. Scale bar, 10 microns.
(TIFF)

S4 Fig Diploid-like SNPs cluster in genomic regions. The location of SNPs (colored circles, with a different color for each chromosome) found via VarScan2 by comparing reads from a non-mutagenized colony of DBY10148 to the S288C reference genome were visualized according to their genomic position. Yeast chromosomes 1 to 16 are represented by a solid black line and are numbered. The mitochondrial genome is denoted as chromosome 17. A red dot approximates the centromeric region.
(TIFF)

S5 Fig Yeast grown in MMS do not have a detectable change in the number of InDels. Same as Fig 3A, except InDels are analyzed here instead of SNPs.
(TIFF)

S6 Fig Characterization of SNPs identified via whole genome sequencing. SNPs identified in Fig 3 were characterized as located between coding sequences (intergenic), causing silent mutations, or causing missense mutations. Neither nonsense (amino acid codon to stop codon) nor nonstop mutations (stop codon to amino acid codon) were identified. Each of the 27 genomes are represented with the day in culture, the mutagen condition, and the replicate ID (A, B, or C). The first yeast genome (Day 0, no mutagen, replicate A) was chosen as the baseline genome to which all the other genomes were compared. The color scheme is the same as in Fig 2A.
(TIFF)

S1 Table Source data. Contains the source data for Figs 3 and S1 (containing data for Fig 2), and S2 Fig.
(XLSX)

S2 Table SNP characterization. Contains the nature, location, and consequence of SNPs identified in Fig 3A. Note that alternative replicates are bolded for ease of reading.
(XLSX)

## Abbreviations

Can-resistant colonies: canavanine-resistant colonies
DI water: deionized water
EDTA: ethylenediaminetetraacetic acid
EtBr: ethidium bromide
GATK: Genomic Analysis Toolkit
HPLC: high-performance liquid chromatography
MMS: methyl methanesulfonate
OD: optical density of yeast culture measured at 600 nm
rpm: rotations per minute
SDS: sodium dodecyl sulfate
SNP: single nucleotide polymorphism
YPD: yeast extract peptone dextrose (yeast media)
