# Supplementary figures and images for: Yeast grown in continuous culture systems can detect mutagens with improved sensitivity relative to the Ames test
Source: PLoS One. 2021 Mar 17;16(3):e0235303. doi: 10.1371/journal.pone.0235303 (PMC7968628; doi:10.1371/journal.pone.0235303)

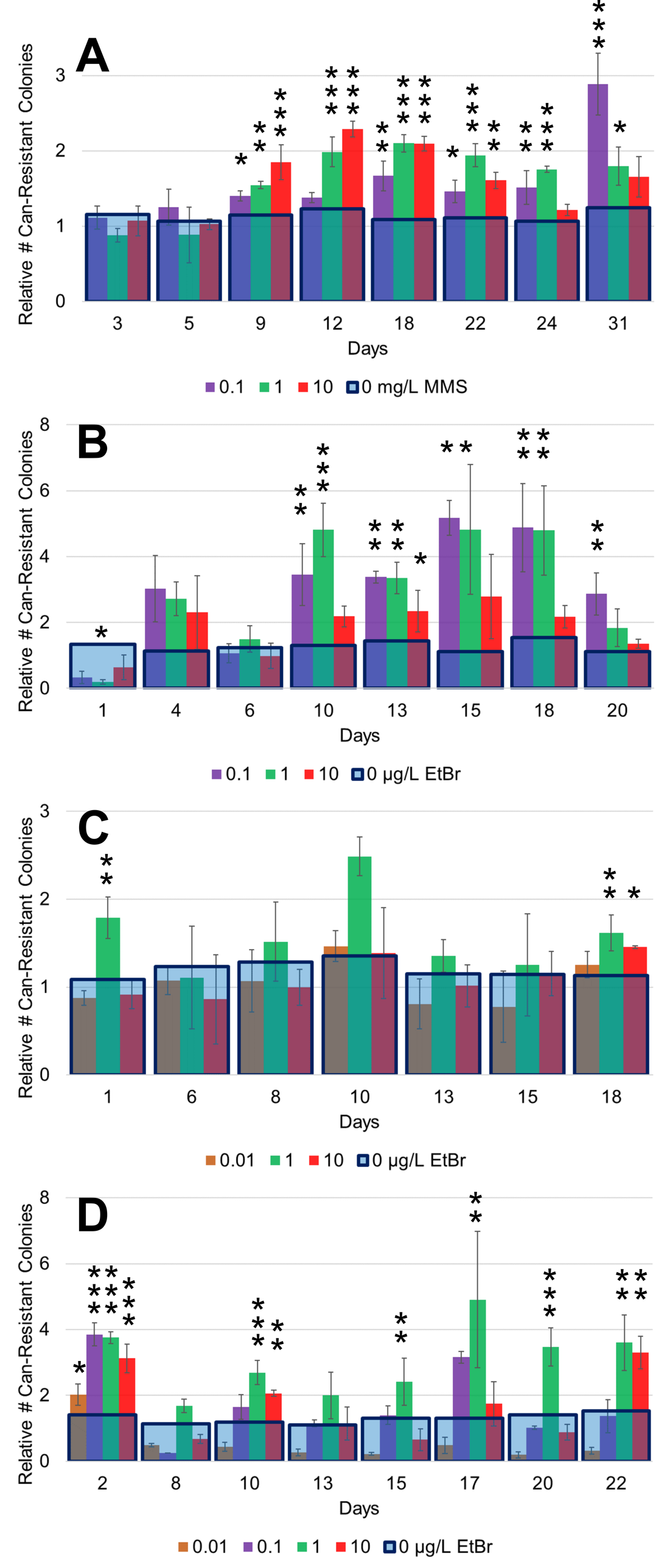

Supplement: S1 Fig — (A) Complete timecourse of MMS chemostats shown in Fig 2A. (B) Complete timecourse of EtBr chemostats shown in Fig 2B. (C, D) Two additional replicates of EtBr chemostats performed in a similar manner to Fig 2B. While yeast grown in chemostats with 0.1, 1.0, and 10 μg EtBr/L consistently produced more canavanine-resistant colonies compared to the no mutagen control, the concentration 0.01 μg EtBr/L was unable to be reliably detected. Moreover, the concentration 1.0 μg EtBr/L consistently produced more canavanine-resistant (Can-resistant) colonies than 10 μg EtBr/L. Data are presented as in Fig 2. A Tukey-HSD test was used to determine statistical significance (* = p < 0.05, ** = p < 0.01, *** = p < 0.001). (TIFF) [file pone.0235303.s001.tiff]

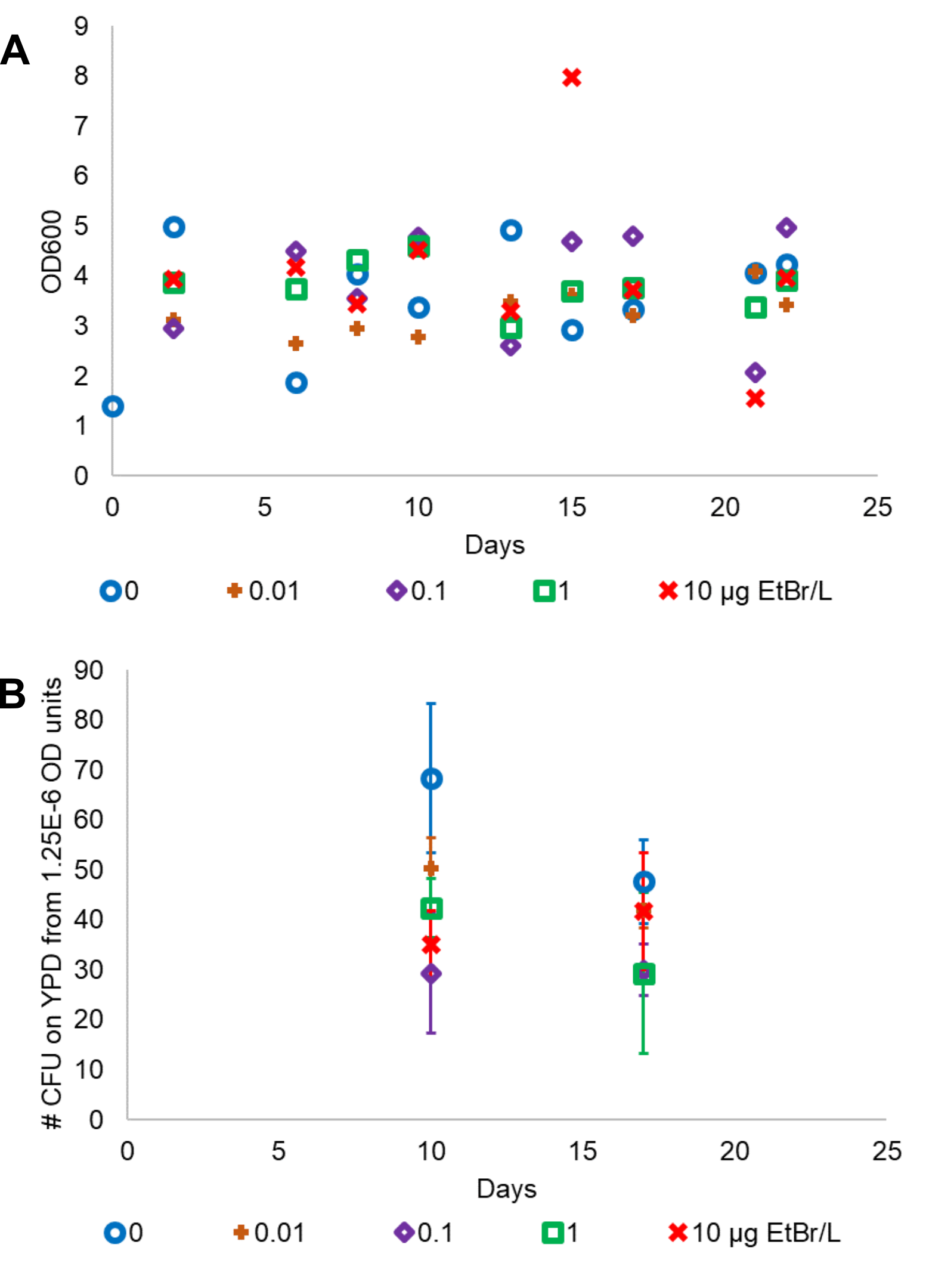

Supplement: S2 Fig — (A) To determine if EtBr had significant toxicity to the yeast, the OD600 of the yeast was assessed over time to identify growth defects and was determined to have no significant variation. (B) To determine if there was any difference in the number of colony forming units, 1.25E-6 OD units of yeast (roughly 40 cells) were obtained by serial dilution and plated onto YPD plates in triplicate. The number of colony forming units (CFU) was counted after 48 hours at 30 °C and is presented as the average of the three trials. The error bars represent standard deviation. The data here correspond to the EtBr chemostats presented in S1D Fig. (TIFF) [file pone.0235303.s002.tiff]

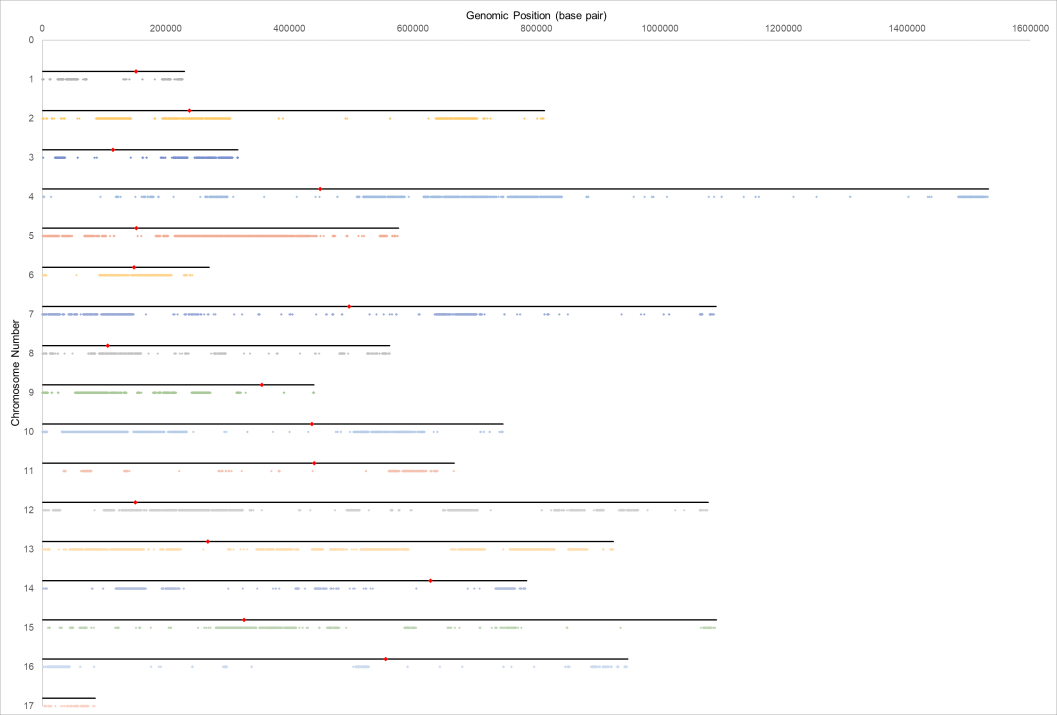

Supplement: S3 Fig — A colony of DBY10148 was used to inoculate an overnight culture of synthetic complete yeast media and allowed to reach late-log phase (OD600 between 2.5 and 3 after about 17 hours of growth at 30 °C). The culture underwent staining with calcofluor white to visualize bud scars. Bud scars were categorized as being either axial (at one pole, expected for haploid yeast) or distal (at both poles, expected for diploid yeast). (A) and (B) are representative images of axial bud scars observed in 96.7% (148/153) of yeast cells analyzed. (C) is a distal bud scar pattern representative of 4.3% (5/153) of yeast cells analyzed. Scale bar, 10 microns. (TIFF) [file pone.0235303.s003.tiff]

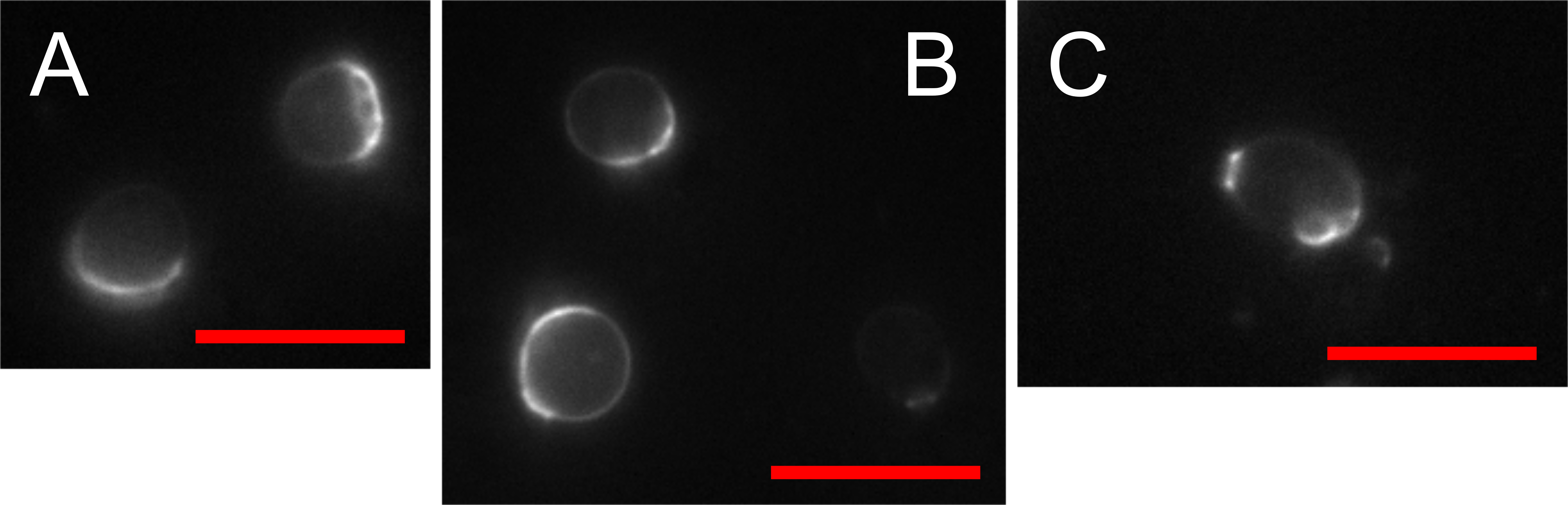

Supplement: S4 Fig — The location of SNPs (colored circles, with a different color for each chromosome) found via VarScan2 by comparing reads from a non-mutagenized colony of DBY10148 to the S288C reference genome were visualized according to their genomic position. Yeast chromosomes 1 to 16 are represented by a solid black line and are numbered. The mitochondrial genome is denoted as chromosome 17. A red dot approximates the centromeric region. (TIFF) [file pone.0235303.s004.tiff]

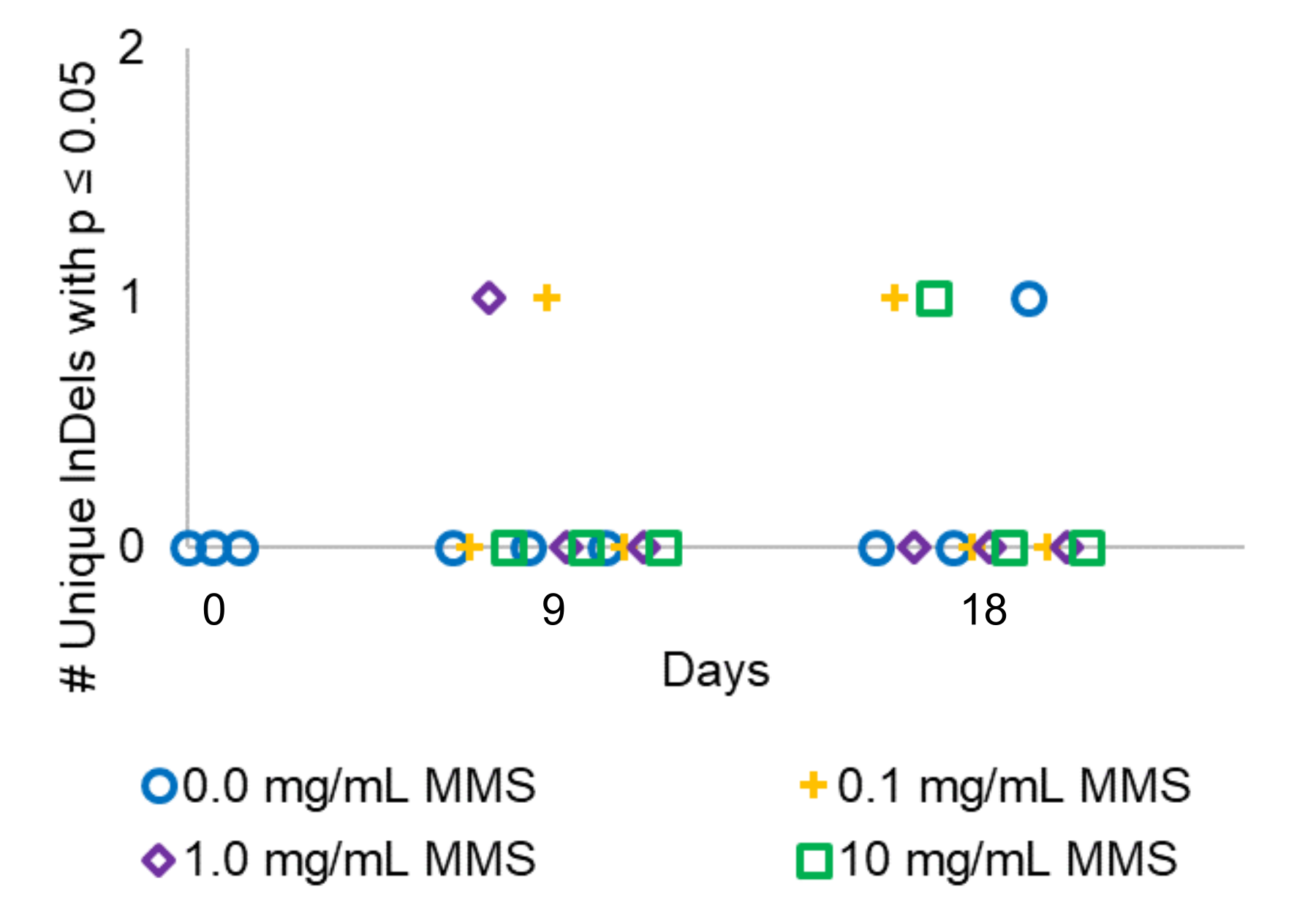

Supplement: S5 Fig — Same as Fig 3A, except InDels are analyzed here instead of SNPs. (TIFF) [file pone.0235303.s005.tiff]

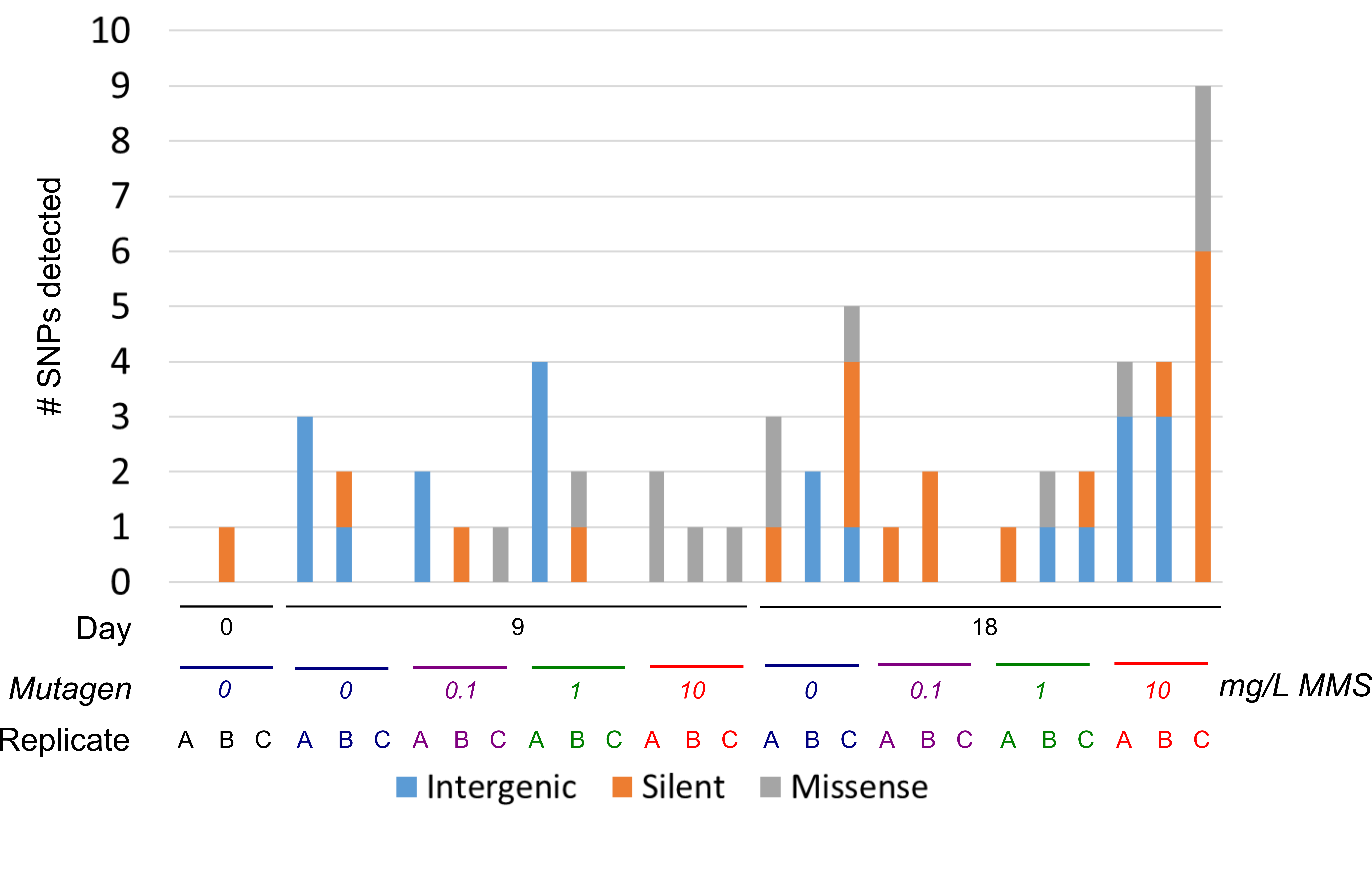

Supplement: S6 Fig — SNPs identified in Fig 3 were characterized as located between coding sequences (intergenic), causing silent mutations, or causing missense mutations. Neither nonsense (amino acid codon to stop codon) nor nonstop mutations (stop codon to amino acid codon) were identified. Each of the 27 genomes are represented with the day in culture, the mutagen condition, and the replicate ID (A, B, or C). The first yeast genome (Day 0, no mutagen, replicate A) was chosen as the baseline genome to which all the other genomes were compared. The color scheme is the same as in Fig 2A. (TIFF) [file pone.0235303.s006.tiff]
